# Comparison of the risk of hospital admission, need for ventilation, sepsis, pneumonitis and death among the recent monkeypox outbreak and historical outbreaks

**DOI:** 10.1186/s12879-023-08599-w

**Published:** 2023-09-18

**Authors:** Florian Gaertner, Saskia Preissner, William Arthur Petri, Olubunmi Atolani, Max Heiland, Susanne Nahles, Robert Preissner, Moritz Hertel

**Affiliations:** 1 Department of Oral and Maxillofacial Surgery, Charité - Universitätsmedizin Berlin, Corporate member of Freie Universität Berlin, Humboldt-Universität zu Berlin and Berlin Institute of Health, Augustenburger Platz 1, 13353 Berlin, Germany; 2 Department of Pathology, University of Virginia, 200 Jeanette Lancaster Way, Charlottesville, VA 22903 USA; 3 Department of Chemistry, University of Ilorin, P.M.B. 1515, Ilorin, 24003 Kwara State Nigeria; 4 Institute of Physiology and Science-IT, Charité - Universitätsmedizin Berlin, Corporate member of Freie Universität Berlin, Humboldt-Universität zu Berlin and Berlin Institute of Health, Philippstr. 12, 10115 Berlin, Germany

**Keywords:** Monkeypox, MPV, MXPV, Zoonosis, Hospital admission, Need for ventilation, sepsis, Pneumonitis, Death

## Abstract

**Background:**

The course of monkeypox can be severe. Our aim was to retrospectively compare the risk of hospital admission, the need for ventilation, sepsis, pneumonitis and death between the recent outbreak and historical outbreaks.

**Materials and Methods:**

Cases of monkeypox were retrieved from the TriNetX database and assigned to either cohort I (recent outbreak between May 1st and September 16th, 2022) and cohort II (historical outbreaks before May 1st, 2022). After matching for age distribution, statistical analysis was performed.

**Results:**

Of 640 patients with monkeypox 81 subjects per cohort remained after matching (mean age±standard deviation = 36.1±18.3 years). Within 56 days after diagnosis 10 patients per cohort were hospitalized (12.4%) and/or developed sepsis (12.4%). The risk of ventilation and pneumonitis were significantly lower among cohort I compared with cohort II (0 vs. 10 cases; risk difference = 12.4%; p = 0.001; Log-Rank test). No cases of death were recorded.

**Conclusion:**

Even though monkeypox provides a risk of severe courses, the infection is self-limiting in most cases. Unlike past outbreaks, the risk of ventilation and pneumonitis may be relatively low among recent outbreaks.

## Introduction

Monkeypox (International Classification of Diseases [ICD-10] code B04) is a zoonotic disease caused by the monkeypox virus (MPV or MXPV). It belongs to the poxviridae family and is closely related to the variola virus (VARV) which causes smallpox. MXPV is a double-strained deoxyribonucleic acid (DNA) virus that replicates inside the cytoplasm of infected mammal cells. Monkeypox can be transmitted via both animal-to-human and human-to-human pathways. Common clinical signs of the disease are fever, pleomorphic rash and lymphadenopathy [1]. Besides these typical symptoms, severe and even fatal courses are possible, especially when pneumonitis, encephalitis or sepsis occur [2]. Even though no regularly licensed treatment is currently available, antiviral therapy with brincidofovir, cidofovir and tecovirimat has been described, especially in patients infected with the human immunodeficiency virus (HIV) [3, 4]. However, it needs to be emphasised that these drugs were used off-label in a limited number of individuals. Furthermore, adverse drug reactions (ADRs) were reported in some cases, leading to the cessation of therapy [1]. Despite that, the recent literature provides evidence of protective immunity against monkeypox through smallpox vaccination [5].

Since the recent outbreak started in the United Kingdom in May 2022, a rising number of cases has been confirmed outside of Africa, where monkeypox is an endemic disease in certain western and central regions [6, 7]. With a view to the coronavirus disease 2019 (COVID-19) pandemic, it was feared that the recent outbreak has the potential to spread worldwide [8]. As the virus emerged among different populations across multiple continents and the route of transmission remains partially uncertain, the local spread of undetected infection was suspected [9]. Even though it remains unclear how the recent outbreak started, there is evidence that all cases outside of Africa might go back to a single infected individual [7, 9]. By sequencing viral genomes, it was found that the MXPV responsible for the recent outbreak strongly resembles a strain from western Africa [6, 7]. Its mortality rate was found to be about 1%, which is significantly lower than that of strains from central Africa, showing death rates of approximately 10%, whereby case fatality was reported to be augmented in children as well as in individuals infected with HIV [6, 7, 10, 11]. Accordingly, there is evidence indicating that the recent outbreak may be associated with a relatively low frequency of complications [12, 13]. Thus, it was hypothesized that the risk of hospital admission, the need for ventilation procedures, sepsis, pneumonitis and death was lower among patients from the recent outbreak in comparison with those from outbreaks in the past. The according aim of the current study was to compare the risk of hospital admission, need for ventilation procedures, sepsis, pneumonitis and death among patients with monkeypox within the recent outbreak and historical outbreaks.

## Materials and methods

### Data Source

With aforementioned aim, the TriNetX Global Health Research Network (TriNetX, Cambridge, Massachusetts, USA) was used to gain retrospective data. This provides access to a significant number of medical records. TriNetX is a database that accumulates clinical data from more than 120 health care organisations (HCOs) from 19 countries. The scope of the network is to enable HCOs, contract research institutes and biopharmaceutical companies to access and exchange longitudinal clinical data and provide state-of-the-art data analytics. By January 2022, TriNetX had collected digital clinical records from more than 250 million subjects. The network is well established to research into medical topics of worldwide interest, including the COVID-19 pandemic [14–16].

### Data assessment

On September 16th, 2022, the TriNetX database was accessed. The index event (in terms of the inclusion criterion which furthermore defines the beginning of the time window in which outcome events are recorded) was defined as diagnosis of “monkeypox” (ICD-10 code B04), whereby the eligibility period was limited to the past 20 years. All diagnoses were made via PCR analysis.

### Allocation and matching

Subsequently, the retrieved patients were assigned to the cohorts I (recent outbreak in terms of diagnosis of monkeypox since May 1st 2022 and the access date) and II (historical outbreaks regarding diagnosis of monkeypox before May 2022). Both cohorts were one-to-one propensity-score matched for age distribution in order to mitigate confounder bias, and to replicate randomized conditions as closely as possible.

### Primary and secondary outcomes

The primary outcome was defined as “death”. Furthermore, the secondary outcomes were “hospitalisation” (admission for hospital inpatient services), “ventilation” (ventilation assist and management, initiation of pressure or volume pre-set ventilators for assisted or controlled breathing), “sepsis” and “pneumonitis”. The time window for recording outcomes was defined as 56 days after the index event. Only medical records which covered the complete time-window were provided by TriNetX. A daily time interval was used to record outcome events.

### Statistical analysis

Data analysis was performed using the statistical analysis tools provided by the TriNetX platform. Specifically, Log-Rank test was applied, whereby p≤0.05 was defined as significance threshold.

## Results

### Study population

**Fig. 1:**
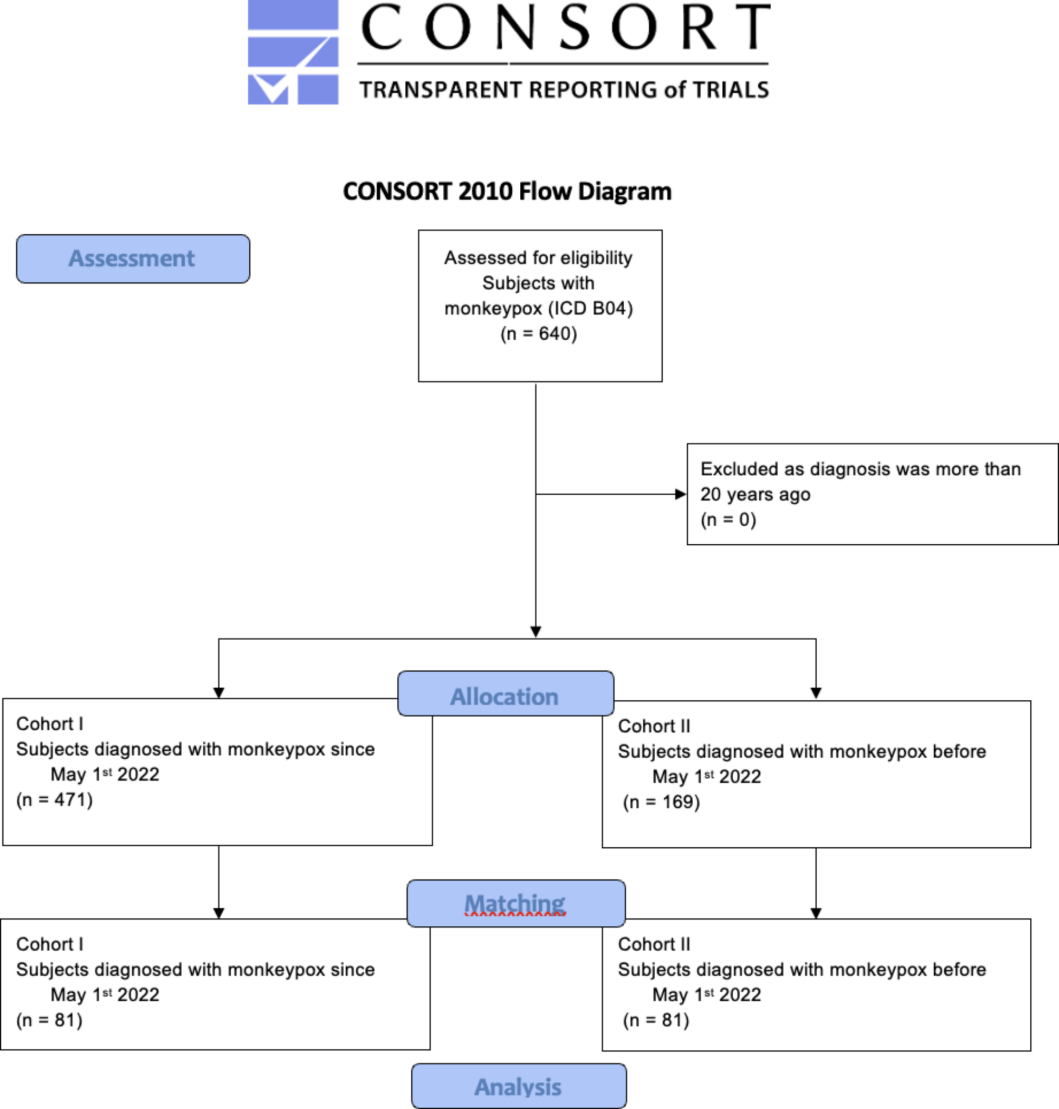
Modified CONSORT-flowchart. ICD = International Classification of Diseases

Initially, 640 patients suffering from monkeypox were obtained from 25 HCOs. Four-hundred-seventy-one (36 females and 435 males) and 169 (99 females and 70 males) individuals were diagnosed with monkeypox since and before May1st 2022, and were thus eligible for the cohorts I and II, respectively. No subject had to be excluded for meeting the index event more than 20 years ago. After propensity-score matching for age distribution each cohort accounted for 81 patients, whereby the mean age did not differ significantly between the cohorts (p = 0.976; Log-Rank test), as shown in Fig. 1. The demographic characteristics of both cohorts before and after matching can be retrieved from Table 1. Before and after matching the proportion of males was significantly higher among cohort I (p<0.001). No information was available regarding the countries in which the cases were recorded.

**Table 1:** Demographic characteristics of the cohorts I (patients diagnosed with monkeypox since May 1st 2022 [“recent outbreak”]) and II (individuals diagnosed with monkeypox before May 2022 [“historical outbreaks”]) before and after matching for age distribution. SD = standard difference

**Before propensity score matching**									
		Cohort			Mean ± SD	N Patients	% of Cohort	P-Value	SD
		I II		Age at Index	35.2 +/- 10.7 55.3 +/- 27.1	471 169	100% 100%	< 0.001	0.976
		I II		Female		36 99	7.6% 58.6%	< 0.001	1.287
		I II		Male		435 70	92.4% 41.4%	< 0.001	1.287
**After propensity score matching**									
		Cohort			Mean ± SD	N Patients	% of Cohort	P-Value	SD
		I II		Age at Index	36.1 +/- 18.3 35.7 +/- 19.1	81 81	100% 100%	0.890	0.022
		I II		Female		13 41	16.0% 50.6%	< 0.001	0.788
		I II		Male		68 40	84.0% 49.4%	< 0.001	0.788

### Primary and secondary outcomes

Among the cohorts I and II, no subject died within 56 days after diagnosis of monkeypox. No statistically significant difference between the cohorts was found regarding the necessity of hospitalization and sepsis. Ten individuals within each cohort were hospitalized (risk of hospitalization = 12.4%), and ten patients in both groups developed sepsis within the investigated time-window (risk of sepsis = 12.4%). Significant differences were found in secondary outcomes ventilation and pneumonitis: Ten individuals of the cases from historical outbreaks required ventilation (risk difference = 12.4%, p = 0.001; Log-Rank test), The risk of pneumonitis was significantly lower among cohort I when compared with cohort II (0 vs. 10 cases; risk difference = 12.4%; p = 0.001; Log-Rank test).

## Discussion

The aim of the present study was to compare the risk of the need for hospital treatment and ventilation procedures, sepsis, pneumonitis and death in subjects diagnosed with monkeypox since May 1st 2020 (cohort I [“recent outbreak”]) and before May 2020 (cohort II [“historical outbreaks”]). As there is literature suggesting that the strain involved in the recent outbreak might provide a lower risk of severe courses [12, 13], the assumption was that the respective risks were lower among patients within the recent outbreak compared to cases from the past. The hypothesis was only partially confirmed. Individuals involved in the recent outbreak showed a significantly lower risk of pneumonitis and ventilation compared with subjects from historical outbreaks. The risk of hospital admission, sepsis and death did not differ significantly within the investigated time-window. Furthermore, the risk of death within 56 days after diagnosis was 0% for both cohorts. This finding is in contradiction with previous reports, where case fatality rates of up to 10% were found [6, 7, 10]. Nevertheless, the mortality among individuals with monkeypox was described to vary between different outbreaks, which was attributed to several factors including the causative MPXV strain [17, 18]. Even though neither information on the involved strain nor on the geographic localization of the identified cases was available, TriNetX predominantly involves HCOs from developed countries, where a high medical standard can be expected. This might explain why no fatal courses were recorded. Despite the risk of fatality, severe but non-lethal courses of monkeypox are possible, which is reflected in the calculated risk of hospital admission of 12.4% for both cohorts. However, ten of the patients needed ventilation procedures in cohort II, and ten cases of sepsis in both cohorts were found within the investigated cohorts. As transmission of monkeypox among the recent outbreak was reported to be associated with men having sex with men [12], the finding that a significantly higher proportion of the infected individuals among cohort I were males is in accordance with the literature.

Regarding the limitations of the present study a certain risk of bias needs to be taken in into consideration. No data on geographic localization was available. As one consequence, the cases within cohort II could not be related to a specific outbreak in the past. Furthermore, neither data on applied therapeutic measures nor on co-morbidities was available, which may especially have influenced the frequency of hospital admission, but also of the other investigated variables. As the vast majority of HCOs within the TriNetX network are located in western high-income countries, all subjects involved in the study should have had access to high standard medical care. It may therefore be carefully assumed that differences in treatment standards are not likely to have caused the observed differences in the risk of ventilation and pneumonitis between both cohorts. Regarding the growing number of cases reported to the World Health Organization (WHO) (41.664 laboratory confirmed cases including 12 deaths since January 1st and as of August 24, 2022) [19], it has to be considered that only a proportion of cases was available for analysis. Future studies might therefore apply a prospective approach in order to investigate if the obtained results can be confirmed.

Despite the fact that monkeypox is the focus of attention due to a recent outbreak in Western countries, it has to be emphasised that the disease is primarily a burden for the African continent, where there have been repeated outbreaks since its description in 1970. Due to the fact that monkeypox is endemic in some of the poorest countries in Western and Central Africa, a relevant proportion of infected individuals does not have access to healthcare services of Western standards, or basic medical care at all [20, 21]. This highlights that the fight against monkeypox should be seen as a challenge to the global community. We conclude that monkeypox incurs a relevant risk of severe courses with the need for hospital treatment. As a consequence, local outbreaks need to be contained using combined measures, including quarantine. Antiviral drugs and vaccines provide potential relief even though no standards have yet been established. Furthermore, we found evidence that the risk of ventilation, pneumonitis might be lower in the recent outbreak compared with historical outbreaks.

## Conclusions

- Monkeypox incurs a relatively high risk of a severe course regarding the need for hospital admission, whereby no difference was found between the recent outbreak and historical outbreaks.
- The risk of ventilation and pneumonitis was found to be lower among patients from the outbreak that started in May 2022 compared with individuals involved in past outbreaks.

## Data Availability

The datasets used and analysed can be retrieved from the TriNetX network (https://trinetx.com). If no access is available, the datasets can be retrieved from the corresponding author upon reasonable request.
